# Evaluation of the Physicochemical Attributes of Beef, Chicken, and Pork Muscles Injected with Microbial Proteases for Designing Senior-Friendly Processed Meat Products

**DOI:** 10.3390/foods13213430

**Published:** 2024-10-28

**Authors:** Si-Young Kim, Dong-Heon Song, Wookyung Chung, Hyun-Shik Choi, Sung Gu Han, Hyun-Wook Kim

**Affiliations:** 1 Department of Food Science and Biotechnology of Animal Resources, Konkuk University, Seoul 05029, Republic of Korea; 2 Food R&D, Samyang Corp., Seongnam 13488, Republic of Korea; wookyung.chung@samyang.com (W.C.); hyunshik.choi@samyang.com (H.-S.C.); 3 Animal Products Utilization Division, National Institute of Animal Science, Rural Development Administration, Wanju 55365, Republic of Korea; timesoul@naver.com; 4 Division of Animal Bioscience & Integrated Biotechnology, Gyeongsang National University, Jinju 52828, Republic of Korea; 5 Department of GreenBio Science, Gyeongsang National University, Jinju 52725, Republic of Korea

**Keywords:** Alcalase, enzymatic hydrolysis, hardness, meat tenderization, myosin heavy chain

## Abstract

In developed countries, the growing elderly population has increased the demand for senior-friendly processed meat products. This study investigated the effects of four commercial microbial proteases (Alcalase, Flavourzyme, Neutrase, and Protamex) on the general physicochemical attributes of beef top round, chicken breast, and pork loin, which are lean muscle cuts suitable for developing senior-friendly meat products. Muscle samples were injected with microbial protease solutions (0.7% and 1.2% (*w*/*w*)), cooked, and used for analysis. The microbial protease injection significantly reduced the hardness of cooked muscles. Despite the evident degradation of the myosin heavy chain in Alcalase treatment, the lowest hardness values were observed in Protamex-treated samples, suggesting that myosin degradation alone does not fully account for tenderness improvement. Unfortunately, microbial protease treatments increased cooking loss in beef and chicken muscles (*p* < 0.05). The surface color characteristics, including redness and yellowness, remained unaffected by the enzymatic treatments, supporting the practical use of these proteases for meat tenderization without inducing color defects. While microbial proteases demonstrate potential for improving meat tenderness, future research should focus on mitigating cooking loss and ensuring desirable taste and flavor for the commercial production of senior-friendly processed meat products using the microbial proteases.

## 1. Introduction

The rapid aging of populations in developed countries has made the development of senior-friendly food products a critical concern within the food industry. Human aging is associated with numerous physiological and functional impairments, particularly affecting food consumption due to dental problems and reduced metabolic capacities [1]. Consequently, the primary objective in designing foods for the elderly is generally to address three key challenges: difficulties in mastication, swallowing, and digestion, while simultaneously meeting their sensory and nutritional requirements. A national survey in Korea revealed that approximately 50% of individuals aged 65 and older experience difficulties with mastication, and only about half maintain a nutritionally balanced diet to support a healthy lifestyle [2]. In response, the Korean Industrial Standard (KS) for Senior-Friendly Foods outlines specific criteria for nutritional content and hardness, categorizing food products based on the level of mastication difficulty. Hardness is classified into three grades: 1st grade for tooth intake (50,000–500,000 N/m^2^), 2nd grade for gum intake (20,000–50,000 N/m^2^), and 3rd grade for tongue intake (below 20,000 N/m^2^) [3]. A recent study reported that the average hardness of 43 commercially available restructured meat products was 64,733 N/m^2^, corresponding to the 1st grade category for tooth intake [4]. However, limited data exist on the hardness of whole-muscle processed meats, such as ham, as most senior-friendly meat products are restructured to achieve the desirable soft texture. Thus, the development of technological innovations that enhance the tenderness of whole-muscle meat products while maintaining their general physicochemical properties is essential for producing senior-friendly meat products.

Meat tenderization is achieved industrially through enzymatic, mechanical, and chemical methods [5], with enzymatic tenderization via exogenous proteases being one of the most effective strategies. This process facilitates the disruption of muscle microstructure and the hydrolysis of muscle proteins, leading to enhanced tenderness [6]. Although endogenous proteases, such as calcium-activated proteinases (e.g., calpains), contribute to meat tenderization during early postmortem proteolysis and aging [7], their abilities vary depending on species and muscle type. Moreover, endogenous proteases exhibit inherent limitations in their maximal efficacy for tenderizing meat [8,9]. Thus, the controlled application of exogenous proteases derived from natural sources, such as papain, bromelain, and ficin, has emerged as an industrially practical solution for tenderizing tough meat [10]. This approach offers advantages in terms of cost-effectiveness and safety, making it particularly suitable for developing meat products tailored to the needs of the elderly. In recent years, microbial proteases from *Bacillus* spp. and *Aspergillus* spp., such as Alcalase, Neutrase, Flavourzyme, and Protamex, have been commercialized and widely used in the food industry for various applications, including enhancing functional properties, reducing food allergens, facilitating the production of fermented foods, and generating bioactive peptides [11]. However, previous research on microbial proteases has predominantly focused on producing and characterizing bioactive protein hydrolysates, with limited exploration of their potential for improving meat tenderness. Additionally, although most processed meat products are formulated with 1–2% salt, primarily sodium chloride (NaCl), the efficacies of microbial proteases on meat tenderization under high ionic strength conditions remain underexplored.

This study was conducted to evaluate the tenderizing effects of commercial microbial proteases (Alcalase, Flavourzyme, Neutrase, and Protamex) on the general physicochemical properties of well-known lean muscle cuts, specifically beef top round, chicken breast, and pork loin, which are characterized by high protein and low-fat content and are considered ideal for developing senior-friendly processed meat products.

## 2. Materials and Methods

### 2.1. Materials

Fresh beef top round (*Musculus adductor*), chicken breast (*M. pectoralis major*), and pork loin (*M. longissimus dorsi*) were purchased from a local market. Commercial microbial proteases, Alcalase (from *Bacillus licheniformis*, EC number 3.4.21.14), Flavourzyme (from *Aspergillus oryzae*, EC number 3.4.11.1), Neutrase (from *Bacillus amyloliquefaciens*, EC number 3.4.24.28), and Protamex (from *Bacillus* sp., EC numbers 3.4.21.62 and 3.4.24.28), were purchased from Novozymes Korea Co., Ltd., Seoul, Republic of Korea. Optimal hydrolysis conditions (pH and temperature) and available strengths of the microbial proteases were obtained from the manufacturers’ online web site as follows: Alcalase (pH 7–9 and 30–65 °C), Flavourzyme (pH 4–8 and 30–65 °C), Neutrase (pH 6–9, 30–65 °C, and 0.8–1.5 AU-N/g), and Protamex (pH 6–9, 30–65 °C, and 1.5 AU-A/g), respectively. All chemicals used were analytic grade.

### 2.2. Sample Prepration

#### 2.2.1. Hydrolytic Efficacy Under Different NaCl Concentrations (Experiment I)

Fresh chicken breast was ground using a meat grinder (MN-22S, Hankook Fugee Industries, Siheung, Republic of Korea) equipped with an 8 mm plate and homogenized with 4 volumes (*v*/*w*) of NaCl solutions, in which the final NaCl concentrations in the homogenate were set at 0, 1, 3, and 5% (*w*/*w*), respectively. The pH of the homogenate was adjusted to pH 8.0 with a few drops of 1 N NaOH solution, and the enzymatic hydrolysis was performed with Alcalase (enzyme to substrate of 1:500) in a 50 °C incubator for 3 h. The reactant was centrifuged at 3000× *g* for 15 min, and the supernatant was collected. The supernatant was stored in a 4 °C refrigerator and used to determine the degree of hydrolysis and protein patterns.

#### 2.2.2. Physicochemical Properties of Microbial Protease-Injected Muscles (Experiment II)

Excessive subcutaneous fat and connective tissue were manually removed from the beef round and pork loin muscles. The trimmed muscles were then weighed and cut into rectangular pieces with an average weight of 110 ± 15 g (*n* = 8/treatment/batch). Chicken breasts (*n* = 8/treatment/batch) were used without additional trimming.

To determine the optimal concentration of microbial proteases for injection, a preliminary experiment was conducted using enzyme concentrations ranging from 0 to 2.0% (*w*/*w*) of the total sample weight, with increments of 0.1% (*w*/*w*). The enzyme solution was manually injected into the prepared muscle samples using a hand-held Dosys^®^ Premium Syringe 174 with a pistol-grip handle (Wheaton, USA). Based on the preliminary observations, the minimum concentration required to achieve measurable changes in hardness was determined to be 0.7% (*w*/*w*), while the maximum concentration to prevent over-tenderization of the surface was set at 1.2% (*w*/*w*).

A full factorial sample arrangement was applied for each muscle type, consisting of 4 enzyme types (Alcalase, Flavourzyme, Neutrase, and Protamex) × 2 enzyme concentrations (0.7% and 1.2%), along with an untreated control. The experiment was repeated in three independent batches on separate days. The pumping rate for all muscle samples was equally set at 120%, meaning that a brine solution equivalent to 20% of the sample weight was injected. No additional ingredients were used, allowing for an isolated assessment of the effects of enzymatic treatment on meat quality. The brine solution was prepared by dissolving the microbial proteases in double-distilled and deionized water (DDW) at concentrations of 0.7% and 1.2% (*w*/*w*) relative to the weight of each muscle sample. The brine solution (20% of the sample weight) was then manually injected into multiple spots on the surface of the prepared beef round, chicken breast, and pork loin muscles using the hand-held syringe. After injection, the samples were individually vacuum-sealed in plastic vacuum bags (PA/PE) and stored at 4 °C overnight to allow for enzyme diffusion and equilibration. On the following day, the samples were cooked in a water bath set at 100 °C for 30 min. This cooking temperature was chosen to ensure the complete inactivation of the injected microbial proteases. After cooking, the samples were cooled to room temperature for 30 min and then used for further physicochemical analysis.

### 2.3. Physicochemical Analysis

#### 2.3.1. Degree of Hydrolysis

The degree of hydrolysis of chicken breast enzymatically hydrolyzed with Alcalase at different NaCl concentrations was determined in triplicate according to the *o*-phtahldialdehyde (OPA) described by Jung and Song [12].

#### 2.3.2. Sodium Dodecyl Sulfate-Polyacrylamide Gel Electrophoresis (SDS-PAGE)

In the preliminary test, the hydrolyzed sample was collected and used for protein profiling. The myofibrillar fraction in microbial protease-injected muscles was isolated according to the method described by Kim et al. [5]. Protein SDS-PAGE was performed according to the method of Laemmli [13], and 4–20% Mini-PROTEAN^®^ precast polyacrylamide gels (#4561095, Bio-Rad Laboratories Inc., Hercules, CA, USA) were used. Thirty microliters (60 μg protein loaded) of each denatured protein sample were loaded onto the gel, and electrophoresis was conducted at 100 V for approximately 2 h. Following electrophoresis, the gel was stained with 0.25% Coomassie Brilliant Blue R-250 solution (B7920, Sigma Chemical Co., St Louis, MO, USA) and subsequently de-stained in a solution composed of methanol–distilled water–acetic acid (50:40:10). The molecular weights of the separated protein bands were compared to a pre-stained protein marker (DokDo-MARK, EBM-1032, Elpisbiotech, Daejeon, Republic of Korea) to compare the molecular weight of specific proteins in the myofibril fraction.

#### 2.3.3. pH Value

The pH values of the cooked sample were measured in triplicate using an insert-type electric pH meter (pH spear, Eutech, Singapore) by inserting the probe directly into the core of the samples. Prior to each measurement, the pH meter was calibrated using standard solutions of pH 4.0, 7.0, and 10.0.

#### 2.3.4. Instrumental Color

The surface color of the cooked sample was measured using a colorimeter (CR-400, Minolta, Osaka, Japan). The measurement setup included an observer size of 8 mm, with illuminant C as the light source. According to the manufacturer’s manual, calibration of the colorimeter was performed using a white tile with reference values (CIE L*: +97.83, CIE a*: −0.43, CIE b*: +1.98). CIE L (lightness), a (redness), and b* (yellowness) values were recorded from five randomly selected spots on the surface of each sample.

#### 2.3.5. Cooking Loss

The cooking loss of the cooked samples was determined by calculating the percentage weight difference between the uncooked and cooked samples using the following equation: Cooking loss (%) = [(weight of uncooked sample (g) − weight of cooked sample (g))/weight of uncooked sample (g)] × 100.

#### 2.3.6. Hardness Measurement

The hardness of the cooked samples was measured following the Korean Industrial Standard for senior-friendly foods, as described by Song et al. [14]. Cooked samples were cut into cube shapes, each measuring 10 mm × 10 mm × 10 mm (8 samples per treatment per batch). Hardness was evaluated using a texture analyzer (CT3, Brookfield Engineering Laboratories, Inc., Middleboro, MA, USA) equipped with a 1 cm diameter probe. The texture profile was generated from twice compressions, with a compression rate set at 70% of the sample height. The test conditions were as follows: pre-test speed of 1 mm/s, test speed of 2 mm/s, and post-test speed of 10 mm/s.

### 2.4. Statistical Analysis

The experimental design followed a completely randomized block design and was conducted in three independent batches. For the preliminary test, a one-way analysis of variance (ANOVA) was performed on the degree of hydrolysis. Duncan’s multiple range test was applied to determine significant differences between treatment means, with significance set at *p* < 0.05. For the analysis of the physicochemical properties of microbial protease-injected and cooked muscles, a two-way ANOVA was conducted using the General Linear Model (GLM) procedure in SPSS 18.0 software (SPSS Inc., Chicago, IL, USA), where enzyme type, concentration, and their interaction effects were considered as main factors. If no significant differences were detected for each effect, the data were pooled and statistically compared to the untreated control using one-way ANOVA or Student’s *t*-test (*p* < 0.05). Significant differences between treatment means were further assessed using Duncan’s multiple range test (*p* < 0.05).

## 3. Results and Discussion

### 3.1. Hydrolytic Efficacy Under Different NaCl Concentrations (Experiment I)

#### 3.1.1. Degree of Hydrolysis

**Figure 1:**
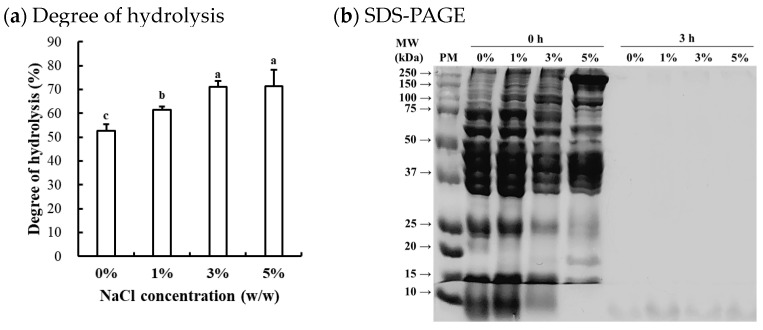
Degree of hydrolysis of Alcalase-hydrolyzed chicken breast at different NaCl concentrations (**a**) and SDS-PAGE (4–20% gradient acrylamide gel) photograph of the soluble fraction from the Alcalase-hydrolyzed chicken breast (**b**). a–c means sharing the same letters between treatments are not significantly different (*p* > 0.05). PM, standard protein marker; 0 h, before enzymatic hydrolysis; 3 h, after 3 h of Alcalase-mediated hydrolysis at 0–5% (*w/w*) NaCl concentrations.

The degree of hydrolysis of enzymatically hydrolyzed chicken breast at different NaCl concentrations is shown in Figure 1a. The degree of hydrolysis was significantly affected by the NaCl concentrations, with the degree of hydrolysis increasing as the NaCl concentration increased (*p* < 0.05). Previous studies on Alcalase-mediated hydrolysis of food proteins have generally been conducted under low ionic strength conditions, as the primary objective has been the production of bioactive peptides [15]. Thus, limited information exists regarding the efficacy of Alcalase hydrolysis under high ionic strength conditions. The results of this study clearly show that Alcalase remains effective even at high ionic strengths. In this regard, the elevated NaCl concentrations may enhance the hydrolysis efficacy by (1) increasing substrate accessibility through protein unfolding, exposing more cleavage sites on the substrate, and/or (2) improving enzyme-substrate interactions by reducing electrostatic repulsions. It is well documented that increasing ionic strength can enhance the solubility and extractability of muscle proteins, particularly salt-soluble myofibrillar proteins [16]. According to Butré et al. [17], moreover, ionic strength influences hydrolysis by altering electrostatic interactions and protein ionization. Increased NaCl concentration could reduce charge repulsion, influencing enzyme-substrate interactions and protein stability. This shifts protein folding and aggregation, affecting cleavage site accessibility and hydrolysis efficiency. Consequently, ionic strength modulates the reaction kinetics and final hydrolysis degree. In this study, however, no significant difference in the degree of hydrolysis between 3% and 5% NaCl concentrations suggests that excessive ionic strength may inhibit Alcalase activity. Claire et al. [17] previously reported that increasing ionic strength from 0 to 0.9 M NaCl had no impact on the hydrolytic efficacy of Alcalase. Given that the NaCl concentration in processed meat production typically ranges from 1 to 2%, it is expected that such levels of NaCl do not adversely affect Alcalase activity on muscle proteins. Similarly, Zago et al. [18] recently found that Alcalase could effectively hydrolyze chicken breast salted with approximately 1.3% NaCl.

#### 3.1.2. SDS-PAGE

The representative SDS-PAGE image (Figure 1b) revealed the typical protein bands of untreated samples (0 h), corresponding to major proteins in chicken breast, including myosin heavy chain (≈200 kDa, MHC), actin (≈42 kDa), and tropomyosin (≈36 kDa), which are consistent with the known protein patterns of chicken breast muscle [18]. The intensity of specific protein bands varied depending on NaCl concentrations, although the overall protein patterns remained similar. As NaCl concentration increased, the intensity of bands associated with salt-soluble myofibrillar proteins became more evident, while the intensity of bands for water-soluble sarcoplasmic proteins decreased. This result could be attributed to the differing solubility and extractability of muscle proteins, as influenced by ionic strength. Similar observations were reported by Xing et al. [19], who found that the addition of 3% NaCl increased the extraction of myosin heavy chains from chicken breast. In this study, after 3 h of Alcalase-mediated hydrolysis, the protein bands disappeared regardless of NaCl concentration, suggesting that most muscle proteins in chicken breast could be hydrolyzed by Alcalase even under high ionic strength conditions. Although hydrolyzed fractions and peptides were not specifically identified, considering that intense hydrolysis occurred at 3% and 5% NaCl concentrations (Figure 1a), our findings suggest that higher NaCl concentrations, within the range typically used in commercial meat products, may enhance the efficacy of Alcalase-mediated hydrolysis in chicken breast by improving the solubility and extractability of myofibrillar proteins.

### 3.2. Physicochemical Properties of Microbial Protease-Injected Muscles (Experiment II)

#### 3.2.1. Protein Patterns of Myofibrillar Proteins from Microbial Protease-Injected Muscles

**Figure 2:**
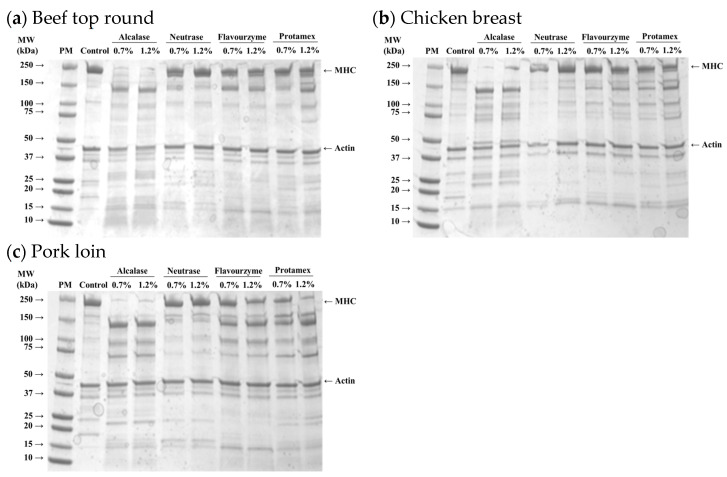
SDS-PAGE (4–20% gradient acrylamide gel) photograph of myofibrils isolated from beef top round (**a**), chicken breast (**b**), and pork loin (**c**) treated with microbial proteases. PM, standard protein marker; Control, untreated muscle sample; MHC, myosin heavy chain.

The protein profiles of myofibrillar fractions isolated from microbial protease-injected muscles are shown in Figure 2. Distinct protein bands representing MHC and actin were observed in all muscle samples. In the protease-injected muscle samples, a notable reduction in the MHC band was evident, along with the emergence of new bands around 25, 70, and 150 kDa, likely corresponding to the degradation products of high molecular weight proteins, including MHC. These changes were most obvious in the Alcalase-treated samples, regardless of muscle type, indicating that Alcalase more efficiently hydrolyzed MHC into lower molecular weight fragments compared to other enzymes. However, the intensity of the actin band remained unchanged despite treatment with microbial enzymes.

Myosin and actin are key proteins responsible for muscle contraction and maintaining the structural integrity of skeletal muscle. Myosin, a major myofibrillar protein, plays a critical role in water-holding capacity and meat tenderness, as it can structurally and chemically entrap water within myofibrils [20]. The degradation of myosin typically improves meat tenderness by weakening the muscle structure [21]. Thus, the findings of this study indicate that Alcalase, among the four microbial proteases tested, was the most effective in degrading MHC across all muscle types. This suggests that the distinct degradation patterns of myofibrillar proteins induced by microbial proteases may lead to significant variations in the physicochemical properties of the protease-injected and cooked muscles. In fact, previous studies have demonstrated that enzymatic degradation of myosin during tenderization processes can enhance the juiciness and tenderness of muscle, thereby improving overall sensory attributes [22,23]. However, in the present study, actin remained largely intact, which was in agreement with the idea that actin might be more resistant to chemical modifications compared to myosin [24].

#### 3.2.2. Effects of Enzyme Type and Concentration on Microbial Protease-Injected Muscles

The statistical analysis of the effects of enzyme type, concentration, and their interaction on the dependent variables (pH, color characteristics, cooking loss, and shear force) of microbial protease-injected and cooked muscles is presented in Table 1. Except for cooking loss, no significant interactions between enzyme type and concentration were observed for the other measured variables across all muscle types. In beef top round, pH, color, cooking loss, and shear force were significantly influenced by enzyme type and/or concentration. In chicken breast, significant changes in cooking loss and shear force were observed following microbial protease injection and cooking. For pork loin, only shear force was significantly affected by enzyme type (*p* = 0.001) among the measured variables. Statistical significance was further evaluated by including an untreated control group to assess the impact of the main effects that caused significant changes in the dependent variables.

#### 3.2.3. pH Value of Microbial Protease-Injected and Cooked Muscles

**Figure 3:**
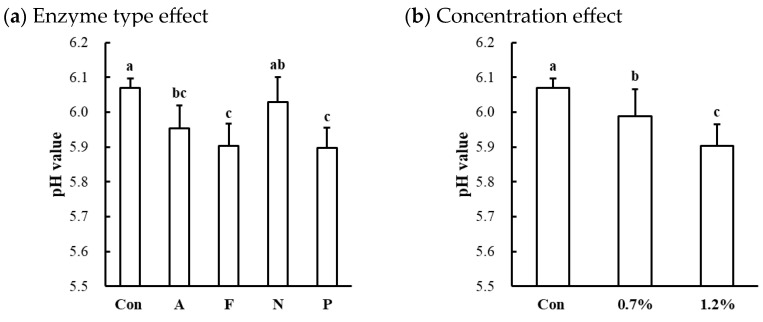
Effects of enzyme type (**a**) and concentration (**b**) on pH value of cooked beef top round muscle. Con, untreated control; A, Alcalase; F, Flavourzyme; N, Neutrase; P, Protamex. a–c means sharing the same letters between treatments within each effect are not significantly different (*p* > 0.05).

Significant effects of enzyme type and concentration on the cooked pH values were observed only in the beef top round (Table 1). Compared to the untreated control, the protease-injected beef top round exhibited a lower cooked pH (*p* < 0.001; Table 2), while no significant differences in cooked pH values were observed between the untreated control and treated samples in chicken breast (6.52 vs. 6.47) and pork loin (6.10 vs. 6.03), respectively. The pH of the raw beef top round used in this study was 5.82, which falls within the normal pH range for beef [25]. Thus, enzymatic tenderization led to a significant decrease in the pH in the beef top round, except for the Neutrase-injected samples (Figure 3a). Additionally, the decrease in pH in the beef top round due to microbial protease injection was significantly dose-dependent (Figure 3b). These findings suggest that enzymatic tenderization using microbial proteases can reduce pH, with the magnitude of this effect varying by muscle type.

The muscle pH is a critical biochemical parameter that influences key meat quality attributes, such as color, water-holding capacity, tenderness, and shelf life [26]. Generally, enzymatic proteolysis by exogenous proteases, which involves the cleavage of peptide bonds in muscle proteins, can release amino acids that may lower the pH of the meat. However, this pH-lowering effect may be muscle-specific, depending on the buffering capacity of different muscle types. According to Puolanne and Kivikari [27], muscles with higher histidine content, such as those composed of white muscle fibers, exhibit greater buffering capacity compared to red muscle fibers. Moreover, the cleavage site specificity and hydrolytic efficiency of the microbial proteases used in this study may further influence the extent of the pH decrease, particularly in beef muscle. In normal muscles, a pH decrease toward the isoelectric point can reduce the net charge and cause protein denaturation, leading to a decrease in water-holding capacity [28]. Thus, in this study, the significant pH reduction in microbial protease-injected beef muscle could negatively affect its water-holding capacity.

#### 3.2.4. Color Characteristic of Microbial Protease-Injected and Cooked Muscles

**Figure 4:**
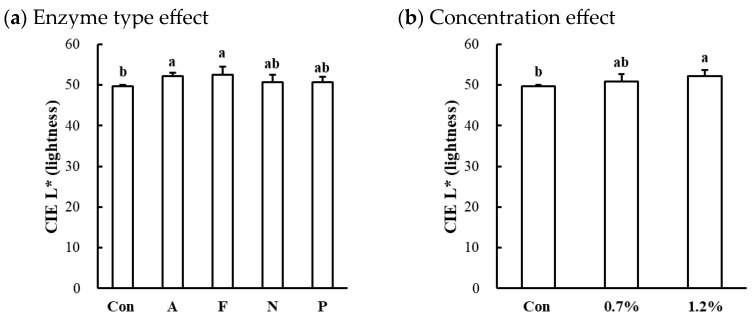
Effects of enzyme type (**a**) and concentration (**b**) on CIE L* (lightness) of microbial protease-injected and cooked beef top round muscle. Con, untreated control; A, Alcalase; F, Flavourzyme; N, Neutrase; P, Protamex. a,b means sharing the same letters between treatments within each effect are not significantly different (*p* > 0.05).

The surface color characteristics of microbial protease-injected and cooked beef, chicken, and pork muscles are shown in Table 3. Significant alterations in CIE L* (lightness) were observed in the beef top round (*p* < 0.001). In the beef muscle, the enzyme type significantly affected lightness, with Alcalase and Flavourzyme treatments resulting in slightly higher lightness values compared to the untreated control (*p* < 0.05; Figure 4a). Additionally, an increase in protease concentration led to an increase in lightness in the beef muscle (*p* < 0.05; Figure 4b). In contrast, no significant differences were observed in CIE a* (redness) or CIE b* (yellowness) across all muscles, regardless of enzyme type or concentration (*p* > 0.05). These results indicate that the application of microbial proteases at the tested concentrations did not adversely affect the surface color attributes of cooked meat, supporting their suitability for meat tenderization without inducing color defects.

Myoglobin is the predominant protein responsible for color changes during the cooking process, as its thermal denaturation leads to a transition from a reddish appearance to a brown-gray color [29]. It has been previously established that myoglobin is resistant to proteolytic degradation by proteases [30], and even it may also function as a protease inhibitor within skeletal muscle (e.g., lysosomal proteases and calcium-dependent proteases) [31]. In this study, no significant changes in redness and yellowness in the protease-injected and cooked samples, which are predominantly influenced by the state of myoglobin, is consistent with these previous observations. The color of processed meat products is a critical quality attribute, particularly in the development of senior-friendly foods, where an appealing and uniform appearance is essential to encourage consumption [1]. Unexpected or heterogeneous color can negatively impact consumer acceptance and limit the practical application of such products. From this perspective, our findings suggest that the injection of microbial proteases does not negatively impact the color characteristics of processed meat, reinforcing their potential suitability for commercial products designed for elderly consumers.

#### 3.2.5. Cooking Loss of Microbial Protease-Injected and Cooked Muscles

**Figure 5:**
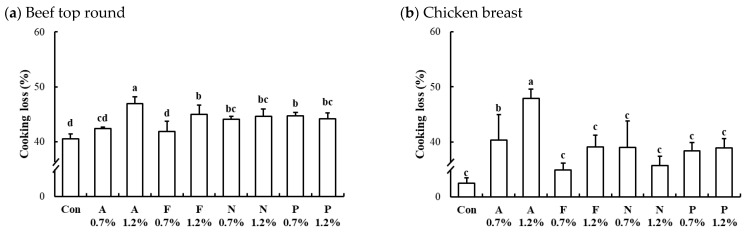
Interaction effects of enzyme type and concentration on cooking loss of microbial protease-injected beef top round (**a**) and chicken breast (**b**). Con, untreated control; A, Alcalase; F, Flavourzyme; N, Neutrase; P, Protamex. The number after protease indicates the applied dose level of the protease (%, *w*/*w*). a–d means sharing the same letters between treatments within each muscle are not significantly different (*p* > 0.05).

The interaction effects of enzyme type and concentration on cooking loss of microbial protease-injected and cooked beef top round and chicken breast are presented in Figure 5. The cooking losses in the untreated controls for beef top round, chicken breast, and pork loin were 40.51%, 32.50%, and 35.24%, respectively. In both beef (Figure 5a) and chicken (Figure 5b) muscles, all protease-injected treatments resulted in significantly higher cooking losses compared to the untreated controls. In contrast, the cooking loss in microbial protease-injected pork loin (37.64%) was comparable to that of the untreated pork loin (*p* > 0.05). For the protease-injected beef and chicken muscles, the treatment with Alcalase at 1.2% (*w*/*w*) exhibited the highest cooking loss, with 46.95% in beef top round and 47.88% in chicken breast (*p* < 0.05). This considerable increase in cooking loss may be attributed to the extensive degradation of myosin, a key myofibrillar protein responsible for water retention [32], as evidenced by the marked reduction of the myosin heavy chain band in Figure 2. As noted earlier, myosin plays a crucial role in the water-holding capacity of muscle tissue, and its breakdown can result in excessive moisture loss during cooking [33].

Cooking loss is a commonly used indicator of the water-holding capacity of meat and is directly linked to key sensory properties such as juiciness and tenderness. The substantial cooking losses observed in this study may also be influenced by the relatively high cooking temperatures used for protease inactivation, as continued proteolytic activity can further compromise muscle structure and water retention. This ongoing hydrolysis may negatively affect the marketability of the final products, particularly during storage and distribution. Our findings clearly demonstrate that enzymatic tenderization using microbial proteases can significantly increase cooking loss in both beef and chicken muscles. The elevated cooking loss, which correlates with reduced moisture content, can adversely affect the mouthfeel of meat, leading to less juicy and favorable sensory properties. Particularly in the protease-injected muscle in this study, where higher cooking temperatures are necessary for enzyme inactivation, the risk of consumer dissatisfaction due to inferior sensory properties may be heightened. Thus, addressing the challenge of improving water-holding capacity is critical for the successful commercialization of protease-injected whole muscle products, especially when targeting the elderly. Moreover, processed meat products with poor water retention capacity may experience continuous moisture loss during distribution and storage, leading to potential quality defects. In this regard, further sensory evaluation by a panel of elderly consumers regarding the sensory characteristics of the final product during storage should be conducted.

#### 3.2.6. Hardness of Microbial Protease-Injected and Cooked Muscles

**Figure 6:**
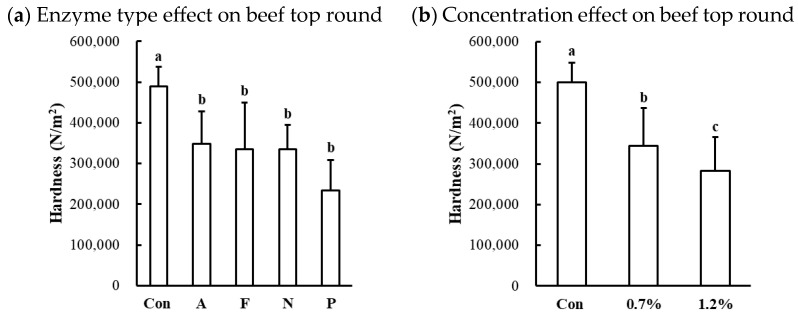

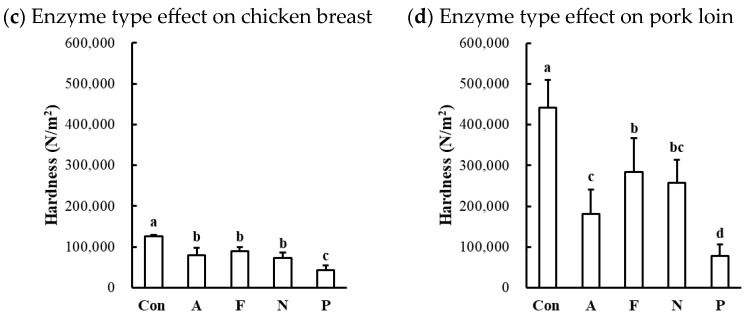
Effects of enzyme type and concentration on hardness of microbial protease-injected and cooked beef top round (**a**,**b**), chicken breast (**c**), and pork loin (**d**). Con, untreated control; A, Alcalase; F, Flavourzyme; N, Neutrase; P, Protamex. a–d means sharing the same letters between treatments within each effect are not significantly different (*p* > 0.05).

The hardness of protease-injected and cooked muscles was significantly affected by enzyme type (Table 1). Compared to the untreated control, all microbial protease-injected treatments exhibited significantly lower hardness values across all muscle types (Figure 6a,c,d). Contrary to our expectations based on the SDS-PAGE results (Figure 2) and cooking loss data (Figure 5), indicating noticeable changes in Alcalase treatment, the lowest hardness values were observed in the Protamex-treated samples for all muscle types. Additionally, the hardness of beef muscles decreased in a dose-dependent manner (Figure 6b). According to the hardness standards of the Korean Industrial Standard (KS) for Senior-Friendly Foods [3], the hardness of untreated beef top round (489,526 N/m^2^) and pork loin (441,692 N/m^2^) was near the upper limit of the 1st grade for tooth intake (50,000–500,000 N/m^2^). Thus, our data suggest that conventionally cooked beef top round and pork loin may not be suitable for elderly consumption. In this study, enzymatic tenderization using four different microbial proteases effectively reduced the hardness of cooked muscles, with the Protamex treatment demonstrating the greatest efficacy. Protamex is a mixture of endo- and exo-proteases derived from *Bacillus subtilis* [34]. Thus, it remains unclear whether the reduction in muscle hardness is specifically due to the interaction between the protease and muscle proteins leading to significant degradation of muscle tissue structure. Even though the fact that the lowest hardness was not observed in the Alcalase-treated samples, despite clear degradation of MHC, suggests that myosin degradation alone cannot fully account for effective muscle tenderization.

## 4. Conclusions

This study demonstrates the effectiveness of four commercial microbial proteases (Alcalase, Flavourzyme, Neutrase, and Protamex) in tenderizing beef top round, chicken breast, and pork loin, which are valuable lean cuts for producing senior-friendly processed meat products. All microbial protease treatments effectively reduced the hardness of cooked muscles. Although Alcalase effectively degraded MHC, the lowest hardness values were observed in the Protamex-treated samples, suggesting that myosin hydrolysis alone does not fully account for muscle tenderization. Additionally, the protease treatments significantly increased cooking loss in the beef and chicken muscles, particularly at higher enzyme concentrations. The surface color characteristics, including redness and yellowness, remained unaffected by the enzymatic treatments, supporting the use of those microbial proteases for meat tenderization without inducing color defects.

Taken together, despite the potential for lowering the hardness of the muscles, concerns were raised regarding sensory quality deterioration due to excessive cooking loss. Furthermore, since the formation of bitter-tasting peptides has been extensively reported as a result of food protein hydrolysis by microbial proteases, further approaches should aim to mitigate cooking loss and ensure desirable taste and flavor for the commercial production of senior-friendly processed meat products using the commercial microbial proteases. More practically, the improvement of water-holding capacity through the addition of hydrocolloids, which have been extensively used in the meat processing industry, could be considered.

## Figures and Tables

**Table 1:** Significance of the effects of enzyme type, concentration, and their interaction on measured variables of microbial protease-injected and cooked muscles.

Muscle Type	Effect	pH	Color Characteristic			Cooking Loss	Shear Force
			CIE L*	CIE a*	CIE b*		
Beef top round	Enzyme type (E)	<0.001	0.029	NS	NS	NS	0.008
	Concentration (C)	<0.001	0.024	NS	NS	0.002	0.009
	Interaction (E × C)	NS ^1^	NS	NS	NS	0.008	NS
Chicken breast	Enzyme type (E)	NS	NS	NS	NS	<0.001	0.001
	Concentration (C)	NS	NS	NS	NS	0.013	NS
	Interaction (E × C)	NS	NS	NS	NS	0.049	NS
Pork loin	Enzyme type (E)	NS	NS	NS	NS	NS	0.001
	Concentration (C)	NS	NS	NS	NS	NS	NS
	Interaction (E × C)	NS	NS	NS	NS	NS	NS

^1^ NS, non-significance (*p* > 0.05).

**Table 2:** The cooked pH value of untreated control and microbial protease-injected (pooled data) muscles.

Treatment	Beef Top Round	Chicken Breast	Pork Loin
Untreated control	6.07 ± 0.03	6.52 ± 0.07	6.10 ± 0.02
Microbial protease-injected	5.95 ± 0.08	6.47 ± 0.06	6.03 ± 0.29
Significance of *p* value	<0.001	NS ^1^	NS

^1^ NS, non-significance (*p* > 0.05).

**Table 3:** Color parameters of the surface on untreated control and microbial protease-injected (pooled data) muscles.

Trait	Treatment	Beef Top Round	Chicken Breast	Pork Loin
CIE L* (lightness)	Untreated control	49.66 ± 0.42	77.76 ± 2.78	73.36 ± 1.50
	Microbial protease-injected	51.52 ± 1.71	79.09 ± 2.61	73.27 ± 1.84
	Significance of *p* value	<0.001	NS ^1^	NS
CIE a* (redness)	Untreated control	4.01 ± 0.76	−0.59 ± 1.29	−2.71 ± 1.15
	Microbial protease-injected	3.82 ± 0.60	−0.99 ± 0.71	−1.62 ± 0.96
	Significance of *p* value	NS	NS	NS
CIE b* (yellowness)	Untreated control	14.54 ± 0.37	14.63 ± 0.64	14.26 ± 0.56
	Microbial protease-injected	14.32 ± 0.51	15.41 ± 0.91	14.58 ± 0.79
	Significance of *p* value	NS	NS	NS

^1^ NS, non-significance (*p* > 0.05).

## Data Availability

The original contributions presented in the study are included in the article; further inquiries can be directed to the corresponding author.
